# Desulfurization of Saudi Arabian crudes by oxidation–extraction method

**DOI:** 10.1007/s13203-015-0112-3

**Published:** 2015-06-06

**Authors:** Raja L. AL Otaibi, Dong Liu, Xulian Hou, Linhua Song, Qingyin Li, Mengfei Li, Hamid O. Almigrin, Zifeng Yan

**Affiliations:** 1 Petrochemical Research Institute, King Abdulaziz City for Science and Technology, PO Box 6086, Riyadh, 11442 Kingdom of Saudi Arabia; 2 State Key Laboratory of Heavy Oil Processing, China University of Petroleum, Qingdao, 266580 China; 3 College of Science, China University of Petroleum, Qingdao, 266580 China; 4 College of Chemical Engineering, Qingdao University of Science and Technology, Qingdao, 266000 China

**Keywords:** Saudi Arabian crudes, Oxidation–extraction, Desulfurization, Fractions

## Abstract

The oxidation–extraction desulfurization of Saudi Arabian crudes was conducted with hydrogen peroxide–acetic acid oxidation system. The selection of extractant, the optimization of oxidation–extraction conditions, and the exploration of desulfurization mechanism were studied. As DMF was used as the extractant, the optimal desulfurization rate of 35.11 % and oil recovery of 95 % were obtained at 70 °C with the molar ratio of peracetic acid to sulfur of 8:1, the molar ratio of acetic acid to hydrogen peroxide of 2:1 and the volume ratio of extractant to oil of 1:1. The desulfurization effect of different fractions in the treated Saudi Arabian crudes was found to obey the following order: gasoline–diesel fraction >VGO fraction >VR fraction, due to different types and structures of sulfur compounds. The oil quality was less affected and most sulfides were mainly extracted via DMF.

## Introduction

With rapid development of economy and technology and increasing demand for crude oil exploitation, the content of sulfur-contenting compounds in the crude oil is ever increasing, leading to poor oil quality. The dissolved sulfides, such as hydrogen sulfide, sulfoether, mercaptan, disulfide, and thiophene, may corrode the equipment in the exploitation, transportation and refining process [1, 2], directly affecting the safe production and even polluting the environment. Therefore, the technology of crude oil desulfurization has attracted more and more interests, and the extensive researches mainly on the non-hydrogenation desulfurization (NHDS) are received [3–5]. The ultrasonic oxidation and bacteria desulfurization methods that remove some sulfides of the heavy crude oil are developed by Sulphco U.S Company [6] and Iran Sharif University of Technology [7], respectively. Additionally, the desulfurizer GX ~201D in Changqing desulfurization process could be added into crude oil directly, but it was non-renewable.

At present, the industrial desulfurization processing technologies of crude oil are still immature. Especially, the desulfurization effect on organic sulfur compounds is insignificant. In view of advantages of mild reaction conditions, high selectivity and low cost [8–10], the deep oxidation desulfurization process has received more concern recently which can remove the refractory sulfur compounds significantly. The oxidants commonly used include NO_2_/HNO_3_, O_3_, peroxyacid and tertbutyl hypochlorite [11–13]. H_2_O_2_ (as oxidant) with organic acid (as oxidation accelerator) among them is widely used in light oil desulfurization process but hardly in crude oil [14]. Moreover, there are few researches systematically on the oxidation desulfurization mechanism.

In this paper, the 30 wt% H_2_O_2_–acetic acid oxidation system was utilized to remove the sulfides from the Saudi Arabian crudes. The optimization of oxidation and extraction conditions was studied, including peracetic acid dosage, HAc/H_2_O_2_ ratio, temperature, reaction time, selection of extractant. The desulfurization effect of different fractions in crude oil was also investigated. Additionally, the oxidation extraction mechanism was proposed.

## Experimental

### Materials

A sample of treated crude oil (Sulfur content, 2.5 wt%) was obtained from Saudi Arabia; Hydrogen peroxide (H_2_O_2_, 30 wt%), acetic acid (HAc), *N*, *N*-dimethylformamide(DMF), dimethylsulfoxide(DMSO), ethanol, ethylene glycol and furfural were purchased commercially.

#### Oxidation and extraction of crude oil

The oxidation of Saudi Arabian crudes was conducted in a glass flask with an electric stirrer. An excess amount of 30 wt% H_2_O_2_ and acetic acid was used to oxidize the sulfur compounds thoroughly. First, H_2_O_2_ and HAC were mixed at a certain ratio to prepare the peracetic acid, which was then put into the crude material. Subsequently, the mixture was stirred at the desired temperature for a certain time.

The extraction of Saudi Arabian crudes was conducted in a three-neck glass flask with an electric stirrer. The extractant was added to the oxidation reaction system. The extraction reaction was carried out at 60 °C for 20 min. Thereafter, the treated crude oil was obtained by centrifuging after the mixture was cooled to room temperature. Finally, oil and solvent were separated using a separatory funnel.

#### Analysis

The sulfur content (wt%) of the crude oil was measured by tubular furnace (Shandong Xianke Instruments Co., Ltd. SX_2_-5-1). The desulfurization rate can be calculated by the following equations:

$$\eta \, = \,\frac{\omega 1\, - \,\omega 2}{\omega 1}\, \times \,100\,\%$$

where

*η*: desulfurization rate, %;

*ω*1: sulfur compounds content of crude oil, %;

*ω*2: sulfur compounds content of treated crude oil, %.

The treated crude oil was distillated to obtain gasoline–diesel fraction, VGO fraction (350–450 °C) and VR fraction, respectively. The gasoline–diesel fraction was characterized by GC-PFPD (USA, GC3800). Besides, VGO fraction was analyzed by GC–MS (Agilent Technologies Co., Ltd., GC6890) and VR fraction was separated into saturates, aromatics, resins and asphaltenes to study the reaction mechanism. As to the GC-PFPD analysis, a HP-5HS (30 m × 0.32 m × 0.25 μm) column was initially maintained at 80 °C for 2 min, then the temperature was ramped to 280 °C at 3 °C/min and maintained for 10 min. The temperature of sample injector was set to 320 °C. The carrier gas was high-purity nitrogen, and the flow rate was 1.0 mL/min. Injection volume was 1 μL.

In addition, crude oil before/after desulfurization and oil in the extractant were analyzed by infrared spectrometer (IR 1730) to investigate the effect of the oxidation–extraction system on the oil quality and distribution of the sulfur compounds.

## Results and discussion

### Optimization of oxidation conditions

**Fig. 1:**
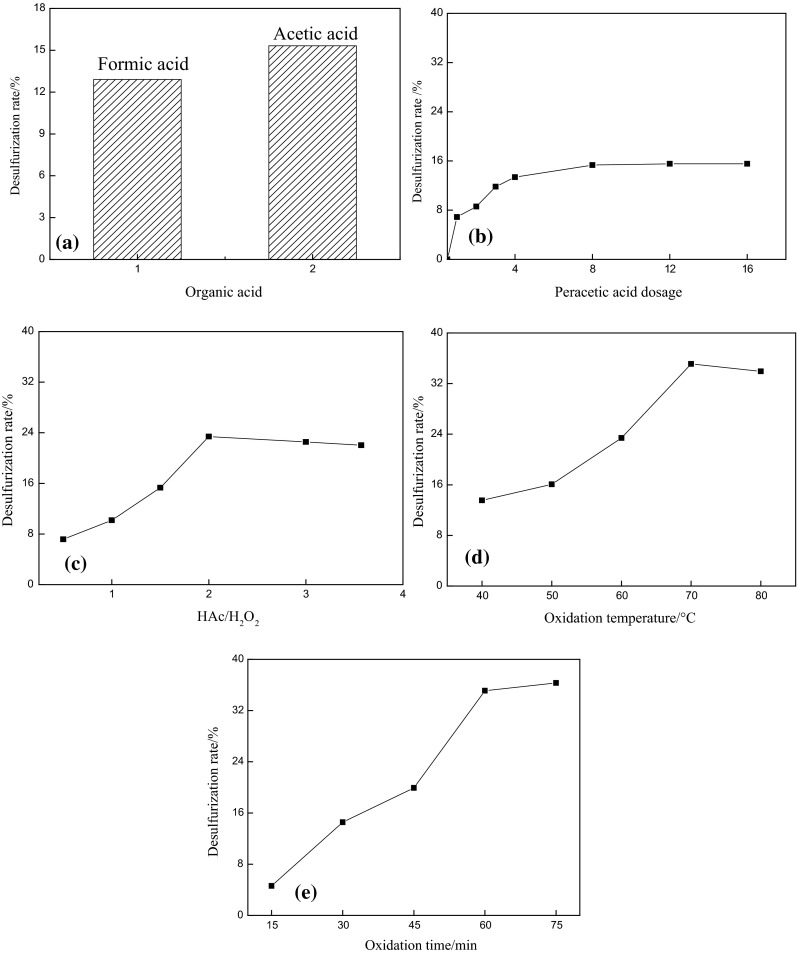
Effect of oxidation conditions on the desulfurization rate. (Peracetic acid dosage is the molar ratio of peracetic acid to sulfur in the oil)

The different oxidation conditions were conducted to study the oxidative desulfurization effect. In this section, the extraction experiment was carried out at 60 °C for 20 min with DMF as extractant, and the extractant to oil volume ratio (E/O) was one. The results are shown in Fig. 1.

H_2_O_2_/organic acid oxidation desulfurization method has been widely investigated and it is a very mature method which can satisfy the purpose of green chemistry in light oil desulfurization process. Otsuki et al. [15] have reported that the sulfur content of oxidized vacuum gas oil was reduced to 0.01 wt% and most of benzothiophene compounds were removed by H_2_O_2_/formic acid oxidation–extraction system.

Figure 1a shows the effect of different organic acids (as oxidation accelerator). The acetic acid was found to have better oxidation-desulfurization effect than formic acid. H_2_O_2_/formic acid system was unable to desulfur effectively due to inherent strong acidity of formic acid. The selectivity in this reaction is much lower under these severe conditions, resulting in sulfides being oxidized incompletely. Additionally, some polar compounds such as the unsaturated hydrocarbons in the crude oil can be also oxidized and extracted simultaneously. To be concluded, on the one hand, the oxidation selectivity for the sulfur-containing compounds and utilization efficiency for H_2_O_2_/organic acid system would be reduced by the strong acidic reaction condition. Thus, a large quantity of oxidant is required and the operation cost is increased. On the other hand, the quality of the crude oil would be changed badly. Clearly, the acidity of acetic acid is weaker than that of formic acid, and then proper oxidation desulfurization of crude oil could be acquired through H_2_O_2_/acetic acid oxidation system. Especially, the resulted oily quality was less affected during the process. The influence of peracetic acid dosage ranging from 0 to 16 was investigated, as the acetic acid/hydroperoxide molar ratio, oxidation temperature and oxidation time were 1.5, 60 °C and 60 min. As shown in Fig. 1b, the desulfurization rate increases initially with the increase of peracetic acid dosage, and then it levels off at 16 %, suggesting that the optimized molar ratio of peracetic acid to sulfur is 8:1.

Effect of HAc/H_2_O_2_ ratio on sulfur removal is depicted in Fig. 1c. Molar ratio of peracetic acid to sulfur is 8:1, oxidation temperature is 60 °C and oxidation time is 60 min. Desulfurization rate presents an uptrend when HAc/H_2_O_2_ ratio is smaller than two and it decreases slightly with HAc/H_2_O_2_ ratio further increased. It was explained that HAc could catalyze reaction as an oxidation accelerator; however, excessive acidity may impair the stability of DMF and weaken extraction effect, leading to reducing the desulfurization rate slightly. Therefore, HAc/H_2_O_2_ ratio of two is chosen.

As illustrated in Fig. 1d, the reaction proceeds completely with the oxidation temperature increased. In particular, the desulfurization rate decreases obviously due to decomposition of H_2_O_2_ and peracetic acid with temperature exceeded 70 °C. Additionally, the unsaturated hydrocarbons and the nitrogen/oxygen-containing compounds are also oxidized, which could destroy the quality of oil and weaken the oxidation selectivity. Therefore, the optimal oxidation temperature is 70 °C.

Figure 1e illustrates that the desulfurization rate elevated obviously with the increasing oxidation time. The oxidation reaction may be completed in 60 min, which was the optimized reaction time.

In conclusion, the optimal oxidation conditions are as follows: H_2_O_2_/acetic acid system is chosen, the molar ratio of peracetic acid to sulfur is 8:1; HAc/H_2_O_2_ molar ratio is 2; the oxidation temperature is 70 °C; the oxidation time is 60 min.

### Optimization of extraction conditions

Thiophenes can be oxidized to sulfones by peracetic acid [16]. In view of atomic structure, sulfur atoms have five more 3d orbits than that of carbon atoms, so they are able to be oxidized easily. Compared with organic sulfur-containing compounds, the generated organic oxides have higher dipole moments, resulting in the increased solubility in DMF, ethanol, DMSO and some other polar solvents. The extraction experiment was carried out under the optimal oxidation condition.

As seen in Table 1, the desulfurization rate of DMF is much higher than that of other solvents at extraction temperature and time of 60 °C and 20 min. Moreover, it could be recovered from atmospheric distillation, thus DMF is selected as suitable extractant.

**Table 1:** Effect of extractant on the desulfurization rate

Extractant	DMF	DMSO	Ethanol	Ethylene glycol	Furfural
Desulfurization rate/%	35.11	20.31	6.92	11.19	–

**Fig. 2:**
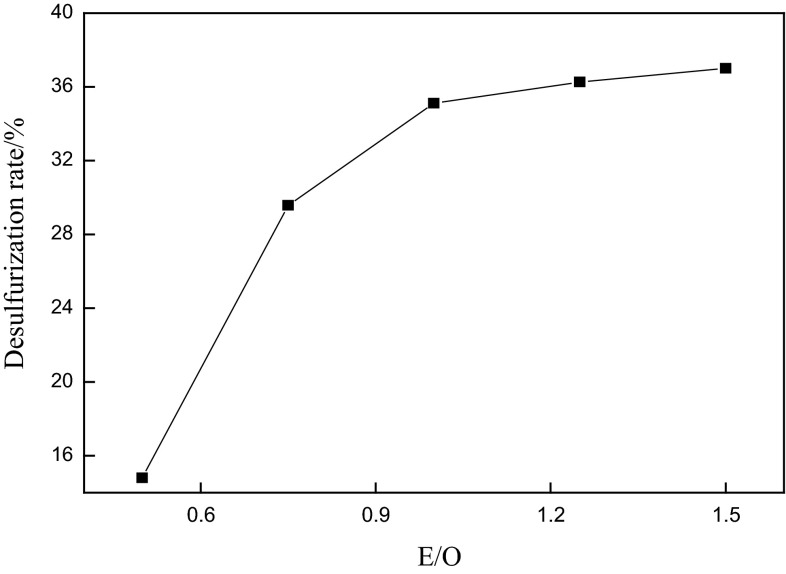
Effect of the extractant to oil volume ratio on the desulfurization rate

Figure 2 shows the influence of the DMF to oil volume ratio (E/O) on the extraction process. The extracted oxide reached to the maximum extent with E/O of 1–1.5. Some polar components could also be extracted as E/O ratio was higher than 1.5, which would reduce the yield of crude oil accordingly. Hence, the chosen E/O ratio was 1, in which the obtained desulfurization rate was up to 35.11 and 95 % of oil could be recovered.

### Mechanism study of the desulfurization effect

#### The yield and desulfurization effect of different fractions

The qualitative analysis on the various sulfides was limited due to the complex composition in the crude oil. Hence, the Saudi Arabian crudes treated from the optimal desulfurization conditions were distilled into three fractions, to further investigate the desulfurization effect on different fractions.

Table 2 shows that different fractions yield remain almost unchanged after oxidative desulfurization, indicating the occurrence of some side reactions during the desulfurization process. In other words, the experimental system has a little influence on the crude oil composition, which makes the process promising for future applications.

**Table 2:** The fractions yield and desulfurization rate

Fractions	Gasoline–diesel	VGO(350–450 °C)	VR
Yield before desulfurization/%	33.47	19.44	47.09
Yield after desulfurization/%	32.28	20.17	47.55
Desulfurization rate/%	59.25	37.58	13.25

The desulfurization rate obeys the following order: gasoline–diesel > VGO > VR. On the one hand, the heavy fraction contains more complicated sulfur compounds with stable structure, suggesting that desulfurization process was in trouble. On the other hand, the components with more strong polarity existed in the heavy fraction could be extracted readily via DMF, which would weaken the sulfur removal effect.

#### GC analysis of gasoline–diesel fraction

Mercaptan belongs to weak acid, and thus can be oxidized easily to disulfide. The alkyl sulfide is a neutral liquid substance, and the disulfide has a poor stability. Both are prone to be oxidized to sulfoxide or sulfonic acid [17], and then removed by extractant. As shown in Table 3, alkylthiophene (96.18 %), benzothiophene (42.95 %) and dibenzothiophene (35.38 %) in gasoline–diesel fraction have been removed after oxidative–extractive reaction.

**Table 3:** Changes of sulfur content in gasoline–diesel fraction

Types of sulfide	Sulfur content/μg g^−1^		Desulfurization rate/%
	Before desulfurization	After desulfurization	
Mercaptan sulfur	196.86	20.24	89.72
Alkyl sulfide	66.68	9.20	86.20
Disulfide	737.32	6.04	99.18
Alkyl thiophene	1754.88	67.00	96.18
BT	2351.62	1341.58	42.95
DBT	2659.64	1718.72	35.38
Others	148.16	62.54	57.79
Total	7915.16	3225.32	59.25

The formula for oxidation desulfurization mechanism is as follows:

As the organic sulfur compounds are oxidized to the corresponding sulfoxide and/or sulfone by peracetic acid, the efficiency of desulfurization can be significantly increased using DMF extraction. Previous researches [15] suggest that the density of electron cloud and space steric hindrance play a critical role in the oxidative desulfurization. The oxidation reaction rate constant *k* increases with the electron cloud density of sulfur atoms. Hence, the reactivity of sulfur compounds is enhanced with the electron density increased for sulfur atom. The effect of oxidative desulfurization should be as follows: dibenzothiophene > benzothiophene > thiophene. However, the experimental results are contrary to the above conclusions. It is speculated that the space steric hindrance may be the dominant factor in the reaction. It should be noted that 59.25 % sulfurs have been removed thoroughly by DMF extraction, indicating that oxidative products in the gasoline–diesel fraction could be extracted effectively to achieve the deep desulfurization goal.

### GC–MS analysis of the VGO fraction

The obtained VGO (350–450 °C) fraction was characterized by GC–MS. The results are listed in Table 4. Some specific sulfides including thioether, benzothiophene, benzonaphthothiophene and benzisothiazole could be identified. After oxidation, these sulfur compounds were reacted to sulfones, and then removed partly after extraction. Especially the di-n-octylsulfide removal rate is the highest, followed by trimethyldibenzothiophene removal rate, while the other sulfide removal rates are relatively low. It is speculated that this is mainly due to the effect of space steric hindrance and inhibition of *N* atoms.

**Table 4:**
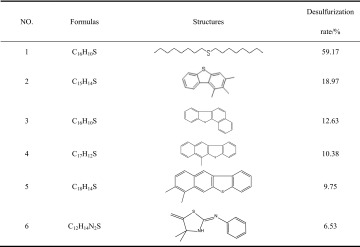
Sulfur compounds’ structures and desulfurization rate in the VGO fraction

The sulfur compounds in the VGO fraction existed mainly in forms of thiophene, benzothiophene, dibenzothiophene compounds and derivatives. Due to the weak polarity in the carbon–carbon bones and carbon–sulfur bones, both organic sulfur-containing species and the corresponding hydrocarbons had the similar polarity. Hence, the sulfur compounds in the crude oil were unable to be removed via polar solvents.

From the molecular perspective, the d orbital electrons were presented in the sulfur atom, so the sulfides were prone to be oxidized selectively to the sulfoxide or sulfone with stronger polarity by peracetic acid. As the oxygen atoms were bonded to the sulfur atoms, dipole moment of the sulfur compounds increased accordingly, and thus their dissolving capacity in the polar solvents (DMF, DMSO, etc.,) was enhanced significantly.

The extraction process was conducted through hydrogen bond interactions between extractant and oxidized compounds, and the selectivity of extractant to the sulfides was vital in the desulfurization process. The desulfurization efficiency was affected greatly by the physicochemical properties of utilized organic solvent, especially polarity. It was shown that the solvent with high polarity may extract the oxidized sulfur compounds (sulfones) effectively, and DMF was considered as the most suitable extractant among these solvents used.

In this study, it was demonstrated that thiophene, benzothiophene, dibenzothiophene compounds and derivatives were oxidized via CH_3_COOOH and extracted through DMF.

#### Four components analysis of the VR fraction

**Fig. 3:**
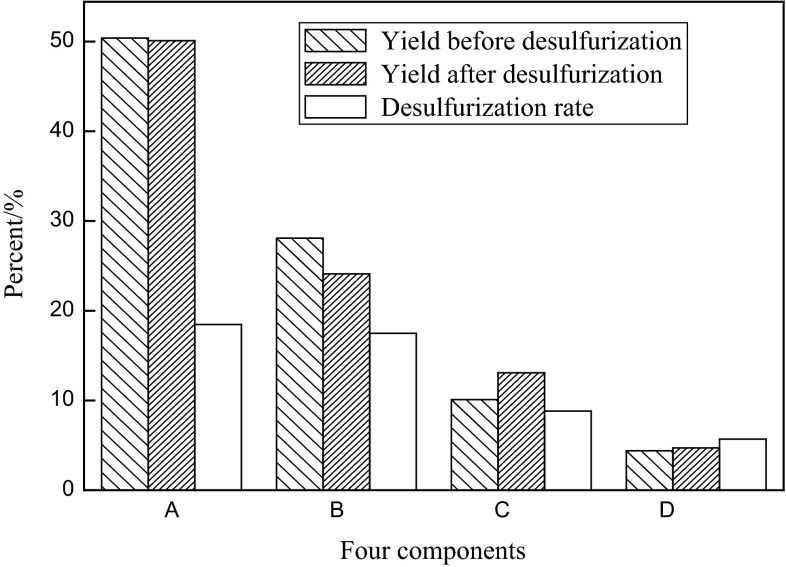
Four components analysis of the VR fraction, *A* saturates, *B* aromatics, *C* resins, *D* asphaltenes

Four components analysis of VR is conducted to investigate the influence of the oxidation–extraction system on the VR fraction compositions. The results (Fig. 3) indicate that the relative yields of saturates and aromatics declined slightly, on the contrary, the yields of resins and asphaltenes increased after desulfurization. Thus, it was preliminarily inferred that some saturates and aromatics were condensed into resins and asphaltenes.

The major sulfides are thioether and thiophene in resins and asphaltenes, such as alkyl thiophene, dibenzothiophene and five- or six-membered ring thioether. In terms of the aromatic heterocyclic compound, thiophene was unable to be removed without efforts due to the stable structure and space steric effect. Desulfurization rate decreases in the following order: saturates > aromatics > resin > asphaltenes. It can be explained that the polarity and condensation degree in the four components increased accordingly. The solubility of sulfides and their corresponding oxidation products—sulfones in the oil were enhanced with the strong polarities, leading to difficult removal.

#### IR analysis of crude oil and the oil in the extractant

**Fig. 4:**
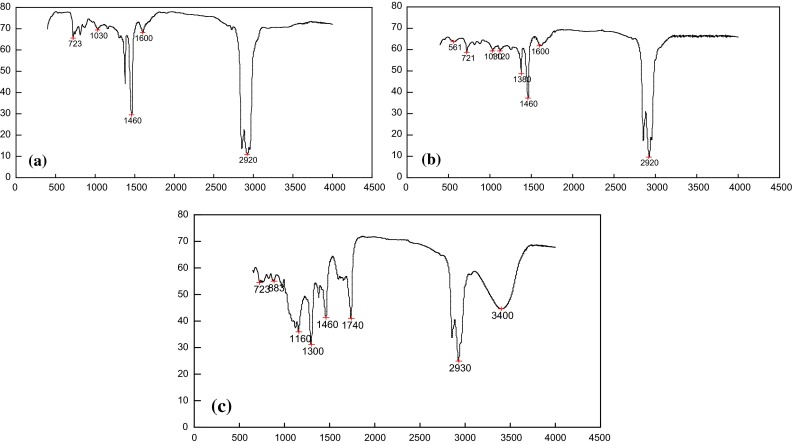
IR analysis of crude oil and extracted oil. **a** Crude oil before desulfurization, **b** crude oil after desulfurization, **c** oil in the extractant

IR spectrums of the crude oil before and after desulfurization are shown in Fig. 4a, b. The results demonstrated that the oily quality hardly changed, indicating the experimental method is feasible.

Compared with raw material (Fig. 4a), oil in the extractant presents two strong absorption peaks at 1300 and 1160 cm^−1^ (Fig. 4c) (DMF and aqueous phase have been removed by distillation), which illustrated that the sulfides have been oxidized to the corresponding sulfones (-SO_2_-). Furthermore, the characteristic adsorption peaks of S=O bond (sulfoxide) and -SO_3_H (sulfonic acid) located at 1030 and 1160 cm^−1^ were obscure. They are probably covered by absorption peaks of -SO_2_-(sulfones) [18]. Besides, 1740 cm^−1^ is attributed to the presence of DMF, mainly caused by the remaining DMF after the distillation; 3400 cm^−1^ represented the absorption peak of vibration hydroxyl group, which may belong to residual moisture or alcohol generated during the oxidation process.

Finally, the sulfur content of the oil in the extractant is determined to be 10.8 % approximately. Obviously, sulfide enrichment can be achieved by extracting the oxidation products from the oxidized oil.

## Conclusions

The oil quality changes a little and most of the sulfides are mainly extracted by DMF after desulfurization. The highest desulfurization rate is obtained at the oxidation temperature of 70 °C for 60 min with a HAc to H_2_O_2_ molar ratio of 2:1 and a peracetic acid to sulfur molar ratio of 8:1. DMF was confirmed to be the best extractant. The optimal extraction condition was considered to be carried out at 60 °C for 20 min with the extractant to oil volume ratio of 1:1. The desulfurization rate of Saudi Arabian crudes could reach as high as 35.11 % and the oil recovery is about 95 %.

The desulfurization effect of different fractions, obtained from the treated Saudi Arabian crudes, is found to obey the following order: gasoline–diesel fraction > VGO fraction > VR fraction.
